# Comparative analysis of EGFR expression in tobacco-related and HPV-associated oral cancer

**DOI:** 10.6026/973206300223310

**Published:** 2026-06-30

**Authors:** Lavanya R.M, Megha Modi, Devashree Shukla, Nandini C., Tamoghna Biswas, Arvind Jain

**Affiliations:** 1 Department of Oral Pathology, Rajiv Gandhi Dental College, Bengaluru, Karnataka, India; 2 Department of Oral and Maxillofacial Surgery, New York University College of Dentistry, New York, United States; 3 Department of Dentistry, LN medical College and JK Hospital, Kolar Road, Bhopal, Madhya Pradesh, India; 4 Department of Oral Pathology, Karnavati School of Dentistry, Karnavati University, Gandhinagar, Gujarat, India; 5 Department of Oral Medicine and Radiology, Saraswati Dental College, Atal Bihari Vajpayee University, Lucknow, Uttar Pradesh, India; 6 Department of Oral Pathology and Microbiology, Maitri College of Dentistry and Research Centre, Durg, Chhattisgarh, India

**Keywords:** Epidermal growth factor receptor (EGFR) expression, tobacco, oral cancer HPV-association, oral squamous cell carcin

## Abstract

Differences in epidermal growth factor receptor (EGFR) expression between tobacco-related and human papillomavirus (HPV)-associated oral squamous cell carcinoma (OSCC) remain insufficiently defined. Therefore, it is of interest to compare EGFR expression in 90 histopathologically confirmed OSCC cases, including tobacco-related OSCC and HPV-associated OSCC. Immunohistochemistry (IHC) was used to assess EGFR expression, while p16 immunostaining served as a surrogate marker for HPV status. Tobacco-related OSCC demonstrated higher EGFR expression than HPV-associated OSCC, indicating differences in tumor biology and therapeutic response. Thus, we show etiological variation in EGFR expression in OSCC, supporting development of targeted and personalized treatment strategies.

## Background:

Oral squamous cell carcinoma (OSCC) is the cause of almost 90 percent of all oral cancer and is a major health issue that poses a problem to a good number of people worldwide. This is especially an issue in countries like India where tobacco has been extensively used in both smoked and smokeless forms, thus causing significant contribution to the prevalence of the diseases [1]. Although there has been an increase in diagnostic modalities and therapeutic interventions, the overall survival of OSCC has not greatly increased over the last decades, in part because of late diagnosis and biological heterogeneity of the disease [2]. Traditionally, OSCC has been strongly associated with environmental risk factors such as tobacco and alcohol consumption. Nitrosamines and polycyclic aromatic hydrocarbons are some of the carcinogens that cause genetic mutations and malignant transformation of oral epithelial cells, which are present in tobacco [3]. With prolonged exposure, there is an accumulation of molecular damage, which causes dysplasia and ultimately carcinoma [4]. Nevertheless, new findings in recent 20 years have pointed to high-risk human papillomavirus (HPV), especially HPV-16, in the etiology of a group of head and neck cancer, including cancers of the mouth [5]. The OSCC of HPV-associated and tobacco-related malignancies is not similar in the context of the molecular pathogenesis and clinical behaviour. Oncoproteins E6 and E7 of the virus facilitate the oncolytic process, which results in carcinogenesis, through the inactivation of tumor suppressor gene p53 and retinoblastoma protein (pRb) and hence interference with the regulation of the cell cycle [6]. It has been believed that these tumors have a better prognosis, increased sensitivity to radiotherapy and different molecular profiles than those caused by tobacco [7]. Epidermal Growth Factor Receptor (EGFR) is a transmembrane glycoprotein that is an ErbB family receptor tyrosine kinase. It is central to controlling vital cellular functions like proliferation, differentiation, angiogenesis, as well as apoptosis [8]. EGFR has been found to be over-expressed in as many as 80-90% of OSCC cases and is linked to tumor aggression, enhanced metastatic potential and unfavorable clinical outcomes [9]. The downstream signaling of EGFR includes RAS-RAF-MAPK and PI3K-AKT activation, which play a role in tumor progression and apoptosis resistance [10]. EGFR expression is seen to vary between tobacco and HPV-associated tumours. Increased expression of EGFR is a common feature of tobacco-associated OSCC as a direct result of carcinogenic activation of oncogenic pathways [11]. Conversely, tumors caused by HPV are more likely to be low EGFR, since oncolytic viruses are activated to cause tumorigenesis by other molecular pathways [12]. This difference has significant therapeutic consequences especially when considering targeted therapies like EGFR inhibitors, which are many times applied in the treatment of head and neck malignancies [13]. Immunohistochemistry of p16 has become a valid substitute marker of carcinogenesis related to HPV. Strong relationships have been found between transcriptionally active HPV infection and overexpression of p16, which is highly utilized in clinical and research environments to distinguish between HPV-related tumours [14]. Therefore, it is of interest to comparatively evaluate etiopathogenesis and molecular expression, EGFR expression in tobacco and HPV-related OSCC in order to improve our knowledge on tumor biology and may lead to individualized treatment options and prognostic indicators.

## Materials and Methodology:

## Study design and setting:

This study was designed as a retrospective comparative observational study conducted in the Department of Oral Pathology and Microbiology of a tertiary care institution.

## Study duration:

The study was conducted over a period of two years, from January 2023 to December 2024.

## Sample size and grouping:

A total of 90 histopathologically confirmed cases of OSCC were included in the study. The cases were equally divided into two groups:

[1] Group I: Tobacco-related OSCC (n = 45)

[2] Group II: HPV-associated OSCC (n = 45)

## Inclusion criteria:

[1] Histopathologically confirmed cases of oral squamous cell carcinoma

[2] Availability of adequate formalin-fixed paraffin-embedded (FFPE) tissue blocks

[3] Documented history of tobacco usage (for Group I)

[4] Positive p16 immunostaining indicating HPV association (for Group II)

## Exclusion criteria:

[1] Recurrent or previously treated OSCC cases

[2] Patients who had received chemotherapy or radiotherapy prior to biopsy

[3] Inadequate or poorly preserved tissue samples

[4] Incomplete clinical or demographic data

## Data collection:

Clinical and demographic details such as age, gender, anatomical site of lesion, tobacco habits and clinical staging were retrieved from institutional archives and patient records.

## Histopathological evaluation:

All cases were reviewed using hematoxylin and eosin (H & E) stained sections to confirm diagnosis and tumor grading according to World Health Organization (WHO) classification.

## Immunohistochemical analysis:

EGFR Immunostaining:

[1] Sections of 4 μm thickness were cut from FFPE tissue blocks.

[2] Antigen retrieval was performed using citrate buffer (pH 6.0) in a microwave oven.

[3] Slides were incubated with primary monoclonal anti-EGFR antibody.

[4] Detection was carried out using a secondary antibody and DAB chromogen.

[5] Slides were counterstained with hematoxylin.

## Evaluation of EGFR expression:

EGFR expression was assessed based on:

[1] Intensity of membranous staining (graded as 0 = none, 1 = mild, 2 = moderate, 3 = strong)

[2] Percentage of positively stained tumor cells

A composite score was derived and cases were categorized into:

[1] Low expression

[2] Moderate expression

[3] High expression

## HPV detection (p16 immunohistochemistry)

[1] p16 immunostaining was used as a surrogate marker for HPV infection.

[2] Strong and diffuse nuclear and cytoplasmic staining in more than 70% of tumor cells was considered positive.

## Statistical analysis:

Data were entered into Microsoft Excel and analyzed using SPSS (IBM Corp., Armonk, NY, USA). Descriptive statistics were expressed as mean ± standard deviation and percentages. Comparison between the two groups was performed using: Chi-square test for categorical variables and Independent t-test for continuous variables. A p-value < 0.05 was considered statistically significant.

## Results:

A total of 90 cases of oral squamous cell carcinoma (OSCC) were evaluated, comprising 45 tobacco-related and 45 HPV-associated cases. High EGFR expression was significantly more prevalent in tobacco-related OSCC (60%) compared to HPV-associated OSCC (22.2%). Conversely, low expression was more common in HPV-associated tumors (37.8%). The difference was statistically significant (p = 0.002), indicating a strong association between carcinogenic pathway and EGFR overexpression (Table 1). High EGFR expression was most frequently observed in moderately differentiated tumors (56.8%), suggesting a correlation with tumor aggressiveness. Poorly differentiated tumors showed predominantly low to moderate expression. The association was statistically significant (p = 0.018) (Table 2). High EGFR expression was significantly associated with lymph node metastasis (63.9%). Patients without metastasis showed predominantly low to moderate expression. This relationship was highly significant (p = 0.001), indicating EGFR as a marker of tumor invasiveness (Table 3).

## Discussion:

The current research revealed that EGFR was found to be much greater in tobacco-related OSCC than HPV-associated OSCC. This finding highlights the role of etiological factors in influencing tumor biology. EGFR has been overexpressed in a high percentage of OSCC cases and linked to tumor progression and poor prognosis. Recently, a study conducted by Gupta *et al.* indicated that about 58% of head and neck cancers were EGFR positive and it was correlated with clinicopathological parameters [1]. Likewise, meta-analysis results reveal that EGFR expression has a strong correlation with lymph node metastasis and poor survival [15]. The EGFR expression was greater in tobacco-related cancers in the current study. This complies with the findings that tobacco carcinogens stimulate EGFR-medicated signaling mechanisms that cause further proliferation and malignant transformation. In a paper by Kaul-Ghanekar *et al.* it was shown that EGFR levels of tobacco users and OSCC patients are higher, which supports its involvement in carcinogenesis [2]. Conversely, HPV-related OSCC was less expressed in EGFR. This is in line with molecular evidence that HPV-initiated carcinogenesis depends on viral oncogenes (E6 and E7) as opposed to EGFR pathway activation. As demonstrated in experimental studies, EGFR engages with HPV oncogenic pathways and perhaps even suppress viral transcription pathways [16]. It was also observed in the present study that there was a significant relationship between EGFR expression and tumor grade. Its expression is higher in moderately differentiated tumors indicating its involvement in tumor progression. The results are in line with other studies that have shown elevated EGFR in more advanced tumors and its association with aggressive clinical phenotypes. Moreover, there existed a close relationship between EGFR expression and lymph node metastasis. This confirms the hypothesis that EGFR enhances tumor invasion, angiogenesis and metastatic potential. Other studies have also reported similar results as EGFR overexpression was associated with involvement of nodal and poor prognosis [11, 14]. The role of EGFR has also been highlighted lately by the developments of targeted therapy. Anti-EGFR agents like cetuximab are commonly used in head and neck cancer but their effectiveness varies according to the HPV status. Tobacco-related tumors that are HPV-negative can respond well to EGFR-targeted therapies as opposed to HPV-positive tumors. In general, the results of the current research align with the existing literature and support the idea that tobacco-associated and HPV-related OSCC can be regarded as different biological entities having different molecular profiles.

## Conclusion:

We show that the expression of EGFR is much greater in oral squamous cell carcinoma caused by tobacco than by HPV. The heightened EGFR expression is also linked to high tumor grade and lymph node metastasis which implies that it is used as an indicator of tumor aggressiveness. Thus, we show that EGFR has the potential to be a valuable prognostic biomarker and can be used to design targeted therapy, especially in tobacco-associated OSCC.

## Figures and Tables

**Table 1:** Comparison of EGFR Expression between study groups

**EGFR Expression**	**Tobacco-related (n=45)**	**HPV-associated (n=45)**	**Total (%)**	**p-value**
Low	5 (11.1%)	17 (37.8%)	22 (24.4%)	
Moderate	13 (28.9%)	18 (40.0%)	31 (34.4%)	
High	27 (60.0%)	10 (22.2%)	37 (41.1%)	0.002

**Table 2:** Association of EGFR Expression with tumor grade

**Tumor Grade**	**Low (%)**	**Moderate (%)**	**High (%)**	**Total**	**p-value**
Well differentiated (n=38)	10 (26.3%)	16 (42.1%)	12 (31.6%)	38	
Moderately differentiated (n=44)	8 (18.2%)	11 (25.0%)	25 (56.8%)	44	
Poorly differentiated (n=8)	4 (50.0%)	4 (50.0%)	0 (0%)	8	0.018

**Table 3:** Association of EGFR Expression with lymph node metastasis

**Lymph Node Status**	**Low (%)**	**Moderate (%)**	**High (%)**	**Total**	**p-value**
Present (n=36)	4 (11.1%)	9 (25.0%)	23 (63.9%)	36	
Absent (n=54)	18 (33.3%)	22 (40.7%)	14 (25.9%)	54	0.001
